# Supplementary material for: Parental-effect gene-drive elements under partial selfing, or why do Caenorhabditis genomes have hyperdivergent regions?
Source: bioRxiv. 2024 Jul 24:2024.07.23.604817. Preprint. [Version 1] doi: 10.1101/2024.07.23.604817 (PMC11291142; doi:10.1101/2024.07.23.604817)

bioRxiv preprint doi: https://doi.org/10.1101/2024.07.23.604817; this version posted July 24, 2024. The copyright holder for this preprint (which was not certified by peer review) is the author/funder, who has granted bioRxiv a license to display the preprint in perpetuity. It is made available under aCC-BY 4.0 International license.

SUPPLEMENTARY FILES

Supplementary text: Recursion equations under androdioecy

Table S1. Comparison of recursion equations for different mating systems.

Figure S1. The effects of *Caenorhabditis*-type androdioecy on genotype and allele-frequency evolution differ between *Medea* and *peel* alleles, and are driven by large differences in heterozygosity between males and hermaphrodites. The four rows show the dynamics for antagonistic *Medeas*, antagonistic peels, a *Medea* invading a population with a resident *peel*, and a *peel* invading a population with a resident *Medea*. The left half of the figure shows results for b =1, and the right half b = 0.5. For each of the eight situations, there are two plots. The left one shows allele frequencies found by iterating the relevant equations (Supplementary File 1) for eleven values of S from 0 to 1, with initial M_1 frequency p = 0.2 and heterozygosity Y = 0. The resident *Medea* M_2 has penetrance k_2 = 0.65 and the invading *Medea* M_1 has penetrance k_1 = 1. The right panel in each pair shows the dynamics in genotype space via De Finetti plot. These examples show again the case of k_1 = 1 and k_2 = 0.65, here with fixed selfing rate S = 0.57. Each trajectory starts from one position at the periphery of the plot and represents the male- or hermaphrodite-specific genotype frequencies through 15 generations. The red line shows the unstable internal equilibrium, conditional on identical starting frequencies for males and females. Under androdioecy, the equilibrium is sensitive to the initial heterozygosity (as seen in its departures from the vertical). To the right of this line, the invading allele fixes, and to the left, it is eliminated. The solid black curve shows genotype frequencies at Hardy-Weinberg equilibrium, and the dashed curve shows genotype frequencies under the neutral extended Hardy Weinberg equilibrium with selfing.

Figure S2. Frequency thresholds for invasion, with b = 0.5. This figure differs from Figure 5 only in the value of b. In an androdioecious population with a resident allele with the specified penetrance (k_2), a completely penetrant allele (k_1=1) can invade and sweep to fixation if its frequency p is above the relevant thick solid line at the indicated selfing rate (S). Below the line, the resident excludes the invader. The dashed lines describe the results for an androdioecious population where the k values are ten-fold lower. For example, the topmost dashed line represents the case of k_2 = 0.095 and k_1 = 0.1.

R package *MedeaFight*. This zipped directory also contains an html guide to using the package and an R script, *PlotMedeaFigures*, that allows for reconstruction of the figures from the manuscript. To use the package, download and unzip the file and then in R, `install.packages("<path to directory>/MedeaFightAndGuide/MedeaFight", repos = NULL, type = "source")`. Finally, `library("MedeaFight")`. For some functions it is necessary to also have the `Ternary` package, which is available from CRAN, at cran.r-project.org.

bioRxiv preprint doi: https://doi.org/10.1101/2024.07.23.604817; this version posted July 24, 2024. The copyright holder for this preprint (which was not certified by peer review) is the author/funder, who has granted bioRxiv a license to display the preprint in perpetuity. It is made available under aCC-BY 4.0 International license.

Supplementary text: Recursion equations under androdioecy

Androdioecy, *Medea*

Taking into account the chromosomal sex determination mechanism in *Caenorhabditis*, each selfing results in hermaphrodite progeny only (XX), while each outcross results in 50% hermaphrodite, 50% male progeny. An outcrossing results in b hermaphrodite progeny for every 1 hermaphrodite that arises from a selfing; b is 0.5 if a cross results in the same number of progeny as a selfing, while it can be greater than 0.5 if a cross yields more progeny than a selfing.

Tracking the frequencies of different matings and the genotypes of their progeny, we can write *Medea* recursions in X, Y, Z, with h and m subscripts to specify hermaphrodite and male values respectively:

Egg-parent	Sperm-parent	Frequency	Surviving Progeny Genotypes: Hermaphrodites			Males		
			M_1M_1	M_1M_2	M_2M_2	M_1M_1	M_1M_2	M_2M_2
M_1M_1		SX_h	1					
M_1M_2		SY_h	$(1-k_2)/4$	$1/2$	$(1-k_1)/4$			
M_2M_2		SZ_h			1			
M_1M_1	M_1M_1	$(1-S)X_hX_m$	b			b		
M_1M_1	M_1M_2	$(1-S)X_hY_m$	$b/2$	$b/2$		$b/2$	$b/2$	
M_1M_1	M_2M_2	$(1-S)X_hZ_m$		b			b	
M_1M_2	M_1M_1	$(1-S)Y_hX_m$	$b(1-k_2)/2$	$b/2$		$b(1-k_2)/2$	$b/2$	
M_1M_2	M_1M_2	$(1-S)Y_hY_m$	$b(1-k_2)/4$	$b/2$	$b(1-k_1)/4$	$b(1-k_2)/4$	$b/2$	$b(1-k_1)/4$
M_1M_2	M_2M_2	$(1-S)Y_hZ_m$		$b/2$	$b(1-k_1)/2$	$b/2$	$b(1-k_1)/2$	
M_2M_2	M_1M_1	$(1-S)Z_hX_m$		b			b	
M_2M_2	M_1M_2	$(1-S)Z_hY_m$		$b/2$	$b/2$		$b/2$	$b/2$
M_2M_2	M_2M_2	$(1-S)Z_hZ_m$			b			b

The recursions for hermaphrodites:

$$X'_h = (S\left(X_h + \left(\frac{1-k_2}{4}\right)Y_h\right) + b(1-S)\left(X_hX_m + \frac{X_hY_m}{2} + \left(\frac{1-k_2}{2}\right)Y_hX_m + \left(\frac{1-k_2}{4}\right)Y_hY_m\right))/\overline{w}_h$$

$$Y'_h = (S\frac{Y_h}{2} + b(1-S)\left(X_hZ_m + \frac{Y_h+Y_m-Y_hY_m}{2} + Z_hX_m\right))/\overline{w}_h$$

$$Z'_h = (S\left(Z_h + \left(\frac{1-k_1}{4}\right)Y_h\right) + b(1-S)\left(Z_hZ_m + \frac{Z_hY_m}{2} + \left(\frac{1-k_1}{2}\right)Y_hZ_m + \left(\frac{1-k_1}{4}\right)Y_hY_m\right))/\overline{w}_h$$

$$\overline{w}_h = S\left(1 - Y_h\left(\frac{k_1+k_2}{4}\right)\right) + b(1-S)\left(1 - \frac{Y_h}{2}\left(X_mk_2 + Y_m\left(\frac{k_1+k_2}{2}\right) + Z_mk_1\right)\right)$$

bioRxiv preprint doi: https://doi.org/10.1101/2024.07.23.604817; this version posted July 24, 2024. The copyright holder for this preprint (which was not certified by peer review) is the author/funder, who has granted bioRxiv a license to display the preprint in perpetuity. It is made available under aCC-BY 4.0 International license.

The recursions for males are simply the outcrossing part of the numerators above, normalized to the male-specific $\overline{w}_m$, which is the outcrossing part of the denominator.

$$X'_m = \left(X_h X_m + \frac{X_h Y_m}{2} + \left(\frac{1-k_2}{2}\right) Y_h X_m + \left(\frac{1-k_2}{4}\right) Y_h Y_m\right))/\overline{w}_m$$
$$Y'_m = \left(X_h Z_m + \frac{Y_h + Y_m - Y_h Y_m}{2} + Z_h X_m\right)/\overline{w}_m$$
$$Z'_m = \left(Z_h Z_m + \frac{Z_h Y_m}{2} + \left(\frac{1-k_1}{2}\right) Y_h Z_m + \left(\frac{1-k_1}{4}\right) Y_h Y_m\right)/\overline{w}_h$$
$$\overline{w}_m = 1 - \frac{Y_h}{2}\left(X_m k_2 + Y_m\left(\frac{k_1+k_2}{2}\right) + Z_m k_1\right)$$

These equations can be consolidated to a system of equations for the four sex-specific allele frequencies p and heterozygosities Y:

$$p'_h = \left(S\left(p_h - \frac{Y_h k_2}{4}\right) + b(1-S)\left(\frac{p_h + p_m - Y_h k_2 p_m}{2}\right)\right)/\overline{w}_h$$
$$Y'_h = \left(S\frac{Y_h}{2} + b(1-S)(p_h q_m + q_h p_m)\right)/\overline{w}_h$$
$$\overline{w}_h = S\left(1 - \frac{Y_h}{2}\left(\frac{k_1+k_2}{2}\right)\right) + b(1-S)\left(1 - \frac{Y_h}{2}(p_m k_2 + q_m k_1)\right)$$
$$p'_m = \left(\frac{p_h + p_m - Y_h k_2 p_m}{2}\right)/\overline{w}_m$$
$$Y'_m = \frac{p_h q_m + q_h p_m}{\overline{w}_m}$$
$$\overline{w}_m = 1 - \frac{Y_h}{2}(p_m k_2 + q_m k_1)$$

These recursions can be rewritten to highlight the effects of the *Medea* alleles, as shown in Table S1:

$$p'_h = \left(p_h\left(S + \frac{b-Sb}{2}\right) + p_m\left(\frac{b-Sb}{2}\right) - \frac{Y_h}{2}k_2\left(\frac{S}{2} + bp_m - Sbp_m\right)\right)/\overline{w}_h$$
$$Y'_h = \left(S\frac{Y_h}{2} + b(1-S)(p_h q_m + q_h p_m)\right)/\overline{w}_h$$
$$\overline{w}_h = S + b - Sb - \frac{Y_h}{2}\left(k_1\left(\frac{S}{2} + bq_m - Sbq_m\right) + k_2\left(\frac{S}{2} + bp_m - Sbp_m\right)\right)$$
$$p'_m = \left(\frac{p_h + p_m - Y_h k_2 p_m}{2}\right)/\overline{w}_m$$
$$Y'_m = \frac{p_h q_m + q_h p_m}{\overline{w}_m}$$
$$\overline{w}_m = 1 - \frac{Y_h}{2}(p_m k_2 + q_m k_1)$$

bioRxiv preprint doi: https://doi.org/10.1101/2024.07.23.604817; this version posted July 24, 2024. The copyright holder for this preprint (which was not certified by peer review) is the author/funder, who has granted bioRxiv a license to display the preprint in perpetuity. It is made available under aCC-BY 4.0 International license.

Androdioecy, *peel*

The mating table below shows the effects of *peel* alleles (P_1 and P_2) in an androdioecious population.

Egg-parent	Sperm-parent	Frequency	Surviving Progeny Genotypes: Hermaphrodites P_1P_1	P_1P_2	P_2P_2	Males P_1P_1	P_1P_2	P_2P_2
P_1P_1		SX_h	1					
P_1P_2		SY_h	$(1-k_2)/4$	$1/2$	$(1-k_1)/4$			
P_2P_2		SZ_h			1			
P_1P_1	P_1P_1	$(1-S)X_hX_P$	b			b		
P_1P_1	P_1P_2	$(1-S)X_hY_P$	$b(1-k_2)/2$	$b/2$		$b(1-k_2)/2$	$b/2$	
P_1P_1	P_2P_2	$(1-S)X_hZ_P$		b			b	
P_1P_2	P_1P_1	$(1-S)Y_hX_P$	$b/2$	$b/2$		$b/2$	$b/2$	
P_1P_2	P_1P_2	$(1-S)Y_hY_P$	$b(1-k_2)/4$	$b/2$	$b(1-k_1)/4$	$b(1-k_2)/4$	$b/2$	$b(1-k_1)/4$
P_1P_2	P_2P_2	$(1-S)Y_hZ_P$		$b/2$	$b/2$		$b/2$	$b/2$
P_2P_2	P_1P_1	$(1-S)Z_hX_P$		b			b	
P_2P_2	P_1P_2	$(1-S)Z_hY_P$		$b/2$	$b(1-k_1)/2$		$b/2$	$b(1-k_1)/2$
P_2P_2	P_2P_2	$(1-S)Z_hZ_P$			b			b

This table yields the following recursions for genotype frequencies:

$$X'_h = (S\left(X_h + \left(\frac{1-k_2}{4}\right)Y_h\right) + b(1-S)\left(X_hX_m + \frac{Y_hX_m}{2} + \left(\frac{1-k_2}{2}\right)X_hY_m + \left(\frac{1-k_2}{4}\right)Y_hY_m\right))/\overline{w}_h$$

$$Y'_h = (S\frac{Y_h}{2} + b(1-S)\left(X_hZ_m + \frac{Y_h + Y_m - Y_hY_m}{2} + Z_hX_m\right))/\overline{w}_h$$

$$Z'_h = (S\left(Z_h + \left(\frac{1-k_1}{4}\right)Y_h\right) + b(1-S)\left(Z_hZ_m + \frac{Y_hZ_m}{2} + \left(\frac{1-k_1}{2}\right)Z_hY_m + \left(\frac{1-k_1}{4}\right)Y_hY_m\right))/\overline{w}_h$$

$$\overline{w}_h = S\left(1 - Y_h\left(\frac{k_1 + k_2}{4}\right)\right) + b(1-S)\left(1 - \frac{Y_m}{2}\left(X_hk_2 + Y_h\left(\frac{k_1+k_2}{2}\right) + Z_hk_1\right)\right)$$

$$X'_m = \left(X_hX_m + \frac{Y_hX_m}{2} + \left(\frac{1-k_2}{2}\right)X_hY_m + \left(\frac{1-k_2}{4}\right)Y_hY_m\right))/\overline{w}_m$$

$$Y'_m = \left(X_hZ_m + \frac{Y_h + Y_m - Y_hY_m}{2} + Z_hX_m\right)/\overline{w}_m$$

$$Z'_m = \left(Z_hZ_m + \frac{Y_hZ_m}{2} + \left(\frac{1-k_1}{2}\right)Z_hY_m + \left(\frac{1-k_1}{4}\right)Y_hY_m\right)/\overline{w}_h$$

$$\overline{w}_m = 1 - \frac{Y_m}{2}(X_hk_2 + Y_h\left(\frac{k_1+k_2}{2}\right) + Z_hk_1)$$

These equations can be consolidated to a system of equations for the four sex-specific allele frequencies p and heterozygosities Y:

$$p'_h = \left(S\left(p_h - \frac{Y_hk_2}{4}\right) + b(1-S)\left(\frac{p_h + p_m - Y_mk_2p_h}{2}\right)\right)/\overline{w}_h$$

$$Y'_h = \left(S\frac{Y_h}{2} + b(1-S)(p_hq_m + q_hp_m)\right)/\overline{w}_h$$

$$\overline{w}_h = S\left(1 - \frac{Y_h}{2}\left(\frac{k_1 + k_2}{2}\right)\right) + b(1-S)\left(1 - \frac{Y_m}{2}(p_hk_2 + q_hk_1)\right)$$

bioRxiv preprint doi: https://doi.org/10.1101/2024.07.23.604817; this version posted July 24, 2024. The copyright holder for this preprint (which was not certified by peer review) is the author/funder, who has granted bioRxiv a license to display the preprint in perpetuity. It is made available under aCC-BY 4.0 International license.

$$p'_m = \left(\frac{p_h + p_m - Y_m k_2 p_h}{2}\right) / \overline{w}_m$$
$$Y'_m = \frac{p_h q_m + q_h p_m}{\overline{w}_m}$$
$$\overline{w}_m = 1 - \frac{Y_m}{2}(p_h k_2 + q_h k_1)$$

And these can be rewritten to highlight the effects of the *peel* alleles, as shown in Table S1:

$$p'_h = \left(p_h\left(S + \frac{b - Sb}{2}\right) + p_m\left(\frac{b - Sb}{2}\right) - \frac{k_2}{2}\left(Y_h\frac{S}{2} + Y_m(bp_h - Sbp_h)\right)\right) / \overline{w}_h$$
$$Y'_h = \left(S\frac{Y_h}{2} + b(1 - S)(p_h q_m + q_h p_m)\right) / \overline{w}_h$$
$$\overline{w}_h = S + b - Sb - \left(\frac{k_1}{2}\left(Y_h\frac{S}{2} + Y_m(bq_h - Sbq_h)\right) + \frac{k_2}{2}\left(Y_h\frac{S}{2} + Y_m(bp_h - Sbp_h)\right)\right)$$
$$p'_m = \left(\frac{p_h + p_m}{2} - \frac{Y_m k_2 p_h}{2}\right) / \overline{w}_m$$
$$Y'_m = \frac{p_h q_m + q_h p_m}{\overline{w}_m}$$
$$\overline{w}_m = 1 - \frac{Y_m}{2}(p_h k_2 + q_h k_1)$$

bioRxiv preprint doi: https://doi.org/10.1101/2024.07.23.604817; this version posted July 24, 2024. The copyright holder for this preprint (which was not certified by peer review) is the author/funder, who has granted bioRxiv a license to display the preprint in perpetuity. It is made available under aCC-BY 4.0 International license.

Androdioecious *peel-Medea* Antagonism

The table below represents the case of antagonistic *Medea* and *peel* alleles (*M* and *P*) in an androdioecious population, where the penetrances of *Medea* and *peel* are k_M and k_P respectively. Note that this model is not symmetrical the way the other models are. Here we let *X, Y,* and *Z* be the genotype frequencies of *Medea* homozygotes, heterozygotes, and *peel* homozygotes, respectively. Consequently, *p* is the *Medea* allele frequency and 1-*p* = *q* is the *peel* allele frequency.

			Surviving Progeny Genotypes					
Herm	Male	Frequency	Hermaphrodites			Males		
			M_1M_1	M_1P_2	P_2P_2	M_1M_1	M_1P_2	P_2P_2
MM		SX_h	1					
MP		SY_h	$(1-k_P)/4$	1/2	$(1-k_M)/4$			
PP		SZ_h			1			
MM	*MM*	$(1-S)X_hX_m$	b			b		
MM	*MP*	$(1-S)X_hY_m$	$b(1-k_P)/2$	$b/2$		$b(1-k_P)/2$	$b/2$	
MM	*PP*	$(1-S)X_hZ_m$		b			b	
MP	*MM*	$(1-S)Y_hX_m$	$b/2$	$b/2$		$b/2$	$b/2$	
MP	*MP*	$(1-S)Y_hY_m$	$b(1-k_P)/4$	$b/2$	$b(1-k_M)/4$	$b(1-k_P)/4$	$b/2$	$b(1-k_M)/4$
MP	*PP*	$(1-S)Y_hZ_m$		$b/2$	$b(1-k_M)/2$		$b/2$	$b(1-k_M)/2$
PP	*MM*	$(1-S)Z_hX_m$		b			b	
PP	*MP*	$(1-S)Z_hY_m$		$b/2$	$b/2$		$b/2$	$b/2$
PP	*PP*	$(1-S)Z_hZ_m$			b			b

This table yields the following recursions for genotype frequencies:

$$X'_h = (S\left(X_h + \left(\frac{1-k_P}{4}\right)Y_h\right) + b(1-S)\left(X_hX_m + \frac{Y_hX_m}{2} + \left(\frac{1-k_P}{2}\right)X_hY_m + \left(\frac{1-k_P}{4}\right)Y_hY_m\right))/\overline{w}_h$$

$$Y'_h = (S\frac{Y_h}{2} + b(1-S)\left(X_hZ_m + \frac{Y_h + Y_m - Y_hY_m}{2} + Z_hX_m\right))/\overline{w}_h$$

$$Z'_h = (S\left(Z_h + \left(\frac{1-k_M}{4}\right)Y_h\right) + b(1-S)\left(Z_hZ_m + \frac{Z_hY_m}{2} + \left(\frac{1-k_M}{2}\right)Y_hZ_m + \left(\frac{1-k_M}{4}\right)Y_hY_m\right))/\overline{w}_h$$

$$\overline{w}_h = S\left(1 - Y_h\left(\frac{k_M + k_P}{4}\right)\right) + b(1-S)\left(1 - \frac{Y_mk_P}{2}\left(X_h + \frac{Y_h}{2}\right) - \frac{Y_hk_M}{2}\left(Z_m + \frac{Y_m}{2}\right)\right)$$

$$X'_m = \left(X_hX_m + \frac{Y_hX_m}{2} + \left(\frac{1-k_P}{2}\right)X_hY_m + \left(\frac{1-k_P}{4}\right)Y_hY_m\right)/\overline{w}_m$$

$$Y'_m = \left(X_hZ_m + \frac{Y_h + Y_m - Y_hY_m}{2} + Z_hX_m\right)/\overline{w}_m$$

$$Z'_m = \left(Z_hZ_m + \frac{Z_hY_m}{2} + \left(\frac{1-k_M}{2}\right)Y_hZ_m + \left(\frac{1-k_M}{4}\right)Y_hY_m\right))/\overline{w}_m$$

$$\overline{w}_m = 1 - \frac{Y_mk_P}{2}\left(X_h + \frac{Y_h}{2}\right) - \frac{Y_hk_M}{2}\left(Z_m + \frac{Y_m}{2}\right)$$

bioRxiv preprint doi: https://doi.org/10.1101/2024.07.23.604817; this version posted July 24, 2024. The copyright holder for this preprint (which was not certified by peer review) is the author/funder, who has granted bioRxiv a license to display the preprint in perpetuity. It is made available under aCC-BY 4.0 International license.

These equations can be consolidated to a system of equations for the four sex-specific allele frequencies p and heterozygosities Y:

$$p'_h = \left(S\left(p_h - \frac{Y_h k_P}{4}\right) + b(1-S)\left(\frac{p_h + p_m - Y_m k_P p_h}{2}\right)\right)/\overline{w}_h$$

$$Y'_h = \left(S\frac{Y_h}{2} + b(1-S)(p_h q_m + q_h p_m)\right)/\overline{w}_h$$

$$\overline{w}_h = S\left(1 - \frac{Y_h}{2}\left(\frac{k_P + k_M}{2}\right)\right) + b(1-S)\left(1 - \left(\frac{Y_m p_h k_P + Y_h q_m k_M}{2}\right)\right)$$

$$p'_m = \left(\frac{p_h + p_m - Y_m k_P p_h}{2}\right)/\overline{w}_m$$

$$Y'_m = \frac{p_h q_m + q_h p_m}{\overline{w}_m}$$

$$\overline{w}_m = 1 - \frac{1}{2}(Y_m k_P p_h + Y_h k_M q_m)$$

And these recursions can be rewritten to highlight the effects of the *Medea* and *peel* alleles, as shown in Table S1:

$$p'_h = \left(p_h\left(S + \frac{b - Sb}{2}\right) + p_m\left(\frac{b - Sb}{2}\right) - \frac{k_P}{2}\left(Y_h\frac{S}{2} + Y_m(bp_h - Sbp_h)\right)\right)/\overline{w}_h$$

$$Y'_h = \left(S\frac{Y_h}{2} + b(1-S)(p_h q_m + q_h p_m)\right)/\overline{w}_h$$

$$\overline{w}_h = S + b - Sb - \left(\frac{k_P}{2}\left(Y_h\frac{S}{2} + Y_m(bp_h - Sbp_h)\right) + \frac{k_M}{2}Y_h\left(\frac{S}{2} + (bq_m - Sbq_m)\right)\right)$$

$$p'_m = \left(\frac{p_h + p_m}{2} - \frac{Y_m k_P p_h}{2}\right)/\overline{w}_m$$

$$Y'_m = \frac{p_h q_m + q_h p_m}{\overline{w}_m}$$

$$\overline{w}_m = 1 - \frac{1}{2}(Y_m k_P p_h + Y_h k_M q_m)$$

Monoecy	Androdioecy, *Medea*	Androdioecy, *peel*	Androdioecy, *Medea* & *peel*
$p' = \frac{\left(p - \frac{Y}{2}k_2\left(\frac{S}{2} + p - Sp\right)\right)}{\bar{w}_h}$<br>$Y' = \left(S\frac{Y}{2} + (1-S)2pq\right)/\bar{w}$<br>$\bar{w} = 1 - \frac{Y}{2}\left(k_1\left(\frac{S}{2} + q - Sq\right) + k_2\left(\frac{S}{2} + p - Sp\right)\right)$	$p'_h = \frac{p_h\left(S + \frac{b-Sb}{2}\right) + p_m\left(\frac{b-Sb}{2}\right) - \frac{Y_h}{2}k_2\left(\frac{S}{2} + bp_m - Sbp_m\right)}{\bar{w}_h}$<br>$Y'_h = \left(S\frac{Y_h}{2} + b(1-S)(p_hq_m + q_hp_m)\right)/\bar{w}_h$<br>$\bar{w}_h = S + b - Sb - \frac{Y_h}{2}\left(k_1\left(\frac{S}{2} + bq_m - Sbq_m\right) + k_2\left(\frac{S}{2} + bp_m - Sbp_m\right)\right)$	$p'_h = \frac{p_h\left(S + \frac{b-Sb}{2}\right) + p_m\left(\frac{b-Sb}{2}\right) - \frac{k_2}{2}\left(Y_h\frac{S}{2} + Y_m(bp_h - Sbp_h)\right)}{\bar{w}_h}$<br>$Y'_h = \left(S\frac{Y_h}{2} + b(1-S)(p_hq_m + q_hp_m)\right)/\bar{w}_h$<br>$\bar{w}_h = S + b - Sb - \left(\frac{k_1}{2}\left(Y_h\frac{S}{2} + Y_m(bq_h - Sbq_h)\right) + \frac{k_2}{2}\left(Y_h\frac{S}{2} + Y_m(bp_h - Sbp_h)\right)\right)$	$p'_h = \frac{p_h\left(S + \frac{b-Sb}{2}\right) + p_m\left(\frac{b-Sb}{2}\right) - \frac{k_P}{2}\left(Y_h\frac{S}{2} + Y_m(bp_h - Sbp_h)\right)}{\bar{w}_h}$<br>$Y'_h = \left(S\frac{Y_h}{2} + b(1-S)(p_hq_m + q_hp_m)\right)/\bar{w}_h$<br>$\bar{w}_h = S + b - Sb - \left(\frac{k_P}{2}\left(Y_h\frac{S}{2} + Y_m(bp_h - Sbp_h)\right) + \frac{k_M}{2}Y_h\left(\frac{S}{2} + (bq_m - Sbq_m)\right)\right)$
Monoecy or Dioecy, obligate outcrossing ($S = 0$)			
$p' = \left(p - \frac{Y}{2}k_2p\right)/\bar{w}$<br>$Y' = 2pq/\bar{w}$<br>$\bar{w} = 1 - \frac{Y}{2}(k_1q + k_2p)$	$p'_m = \left(\frac{p_h + p_m}{2} - \frac{Y_h}{2}k_2p_m\right)/\bar{w}_m$<br>$Y'_m = (p_hq_m + q_hp_m)/\bar{w}_m$<br>$\bar{w}_m = 1 - \frac{Y_h}{2}(k_1q_m + k_2p_m)$	$p'_m = \left(\frac{p_h + p_m}{2} - \frac{Y_m}{2}k_2p_h\right)/\bar{w}_m$<br>$Y'_m = (p_hq_m + q_hp_m)/\bar{w}_m$<br>$\bar{w}_m = 1 - \frac{Y_m}{2}(k_1q_h + k_2p_h)$	$p'_m = \left(\frac{p_h + p_m}{2} - \frac{Y_m}{2}k_Pp_h\right)/\bar{w}_m$<br>$Y'_m = (p_hq_m + q_hp_m)/\bar{w}_m$<br>$\bar{w}_m = 1 - \left(\frac{Y_m}{2}k_Pp_h + \frac{Y_h}{2}k_Mq_m\right)$

Table S1. Comparison of recursion equations for different mating systems.
Sex-specific recursion equations under androdioecy resemble the monoecious case for hermaphrodites and the obligately outcrossing case for males. Important differences are that the allele frequency p is replaced by its sex-weighted average in hermaphrodites and its unweighted average in males, and the generation of heterozygotes by outcrossing, $2pq$ under monoecy or dioecy, is $p_hq_m + q_hp_m$ under androdioecy, influenced by sex differences in allele frequencies. Finally, *Medea* and *peel* elements differ in which sex's heterozygosity and allele frequency influence the effects of the alleles.

Variables and parameters:

p	M_1 allele frequency
q	M_2 allele frequency, 1-p
Y	M_1M_2 heterozygote genotype frequency
$\bar{w}$	Population mean fitness, the proportion of zygotes that survive to reproduce
S	Selfing rate
b	Ratio of hermaphrodite progeny from an outcrossing to hermaphrodite progeny from a selfing
k_1	penetrance of the M_1 allele
k_2	penetrance of the M_2 allele
k_M	penetrance of the *Medea* allele in the *Medea-peel* antagonism model
k_P	penetrance of the *peel* allele in the *Medea-peel* antagonism model
h	subscript for hermaphrodite-specific variables
m	subscript for male-specific variables

bioRxiv preprint doi: https://doi.org/10.1101/2024.07.23.604817; this version posted July 24, 2024. The copyright holder for this preprint (which was not certified by peer review) is the author/funder, who has granted bioRxiv a license to display the preprint in perpetuity. It is made available under aCC-BY 4.0 International license.

Figure S1. Dynamics under androdioecy

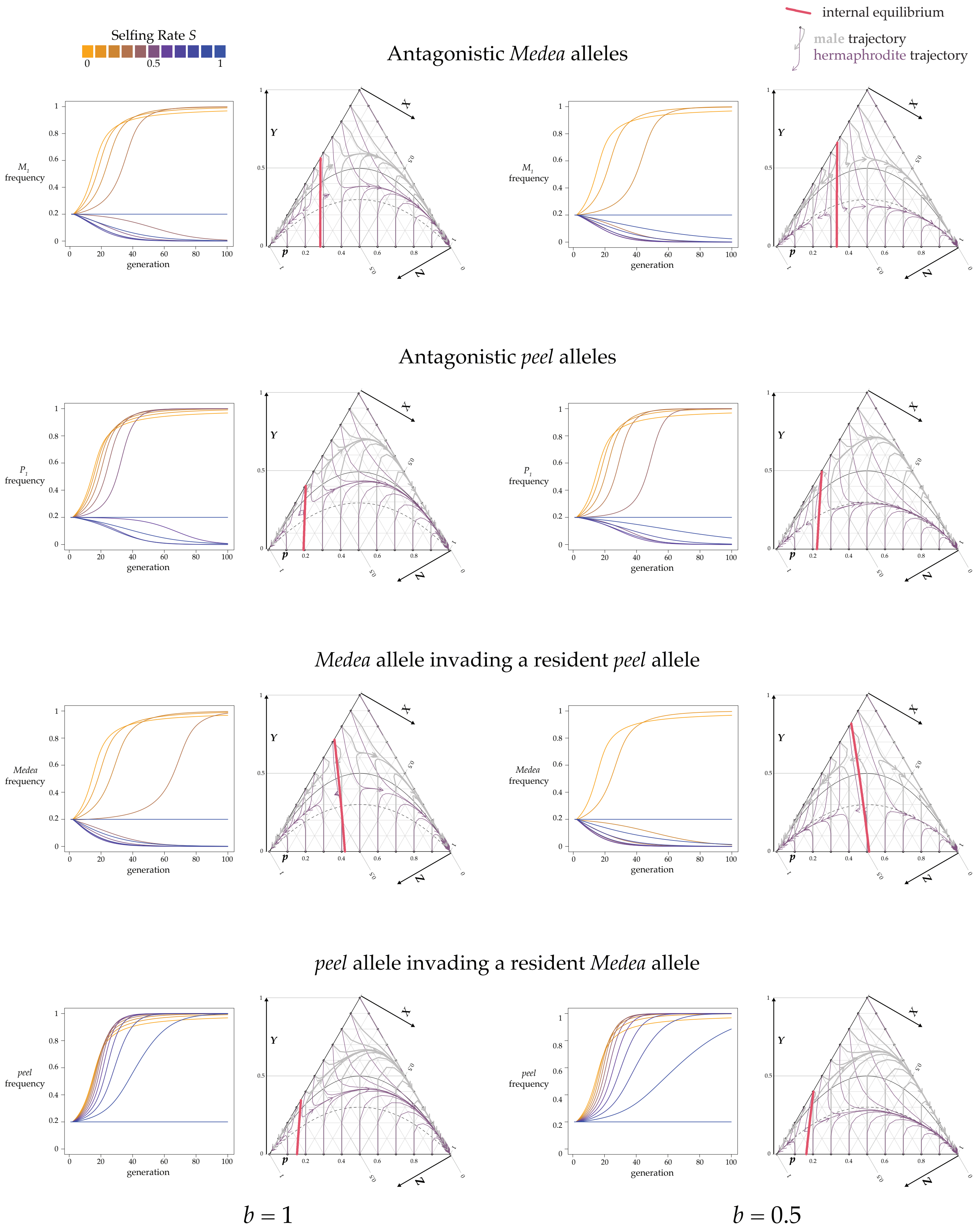

bioRxiv preprint doi: https://doi.org/10.1101/2024.07.23.604817; this version posted July 24, 2024. The copyright holder for this preprint (which was not certified by peer review) is the author/funder, who has granted bioRxiv a license to display the preprint in perpetuity. It is made available under aCC-BY 4.0 International license.

Figure S2. Invasion thresholds with $b = 0.5$

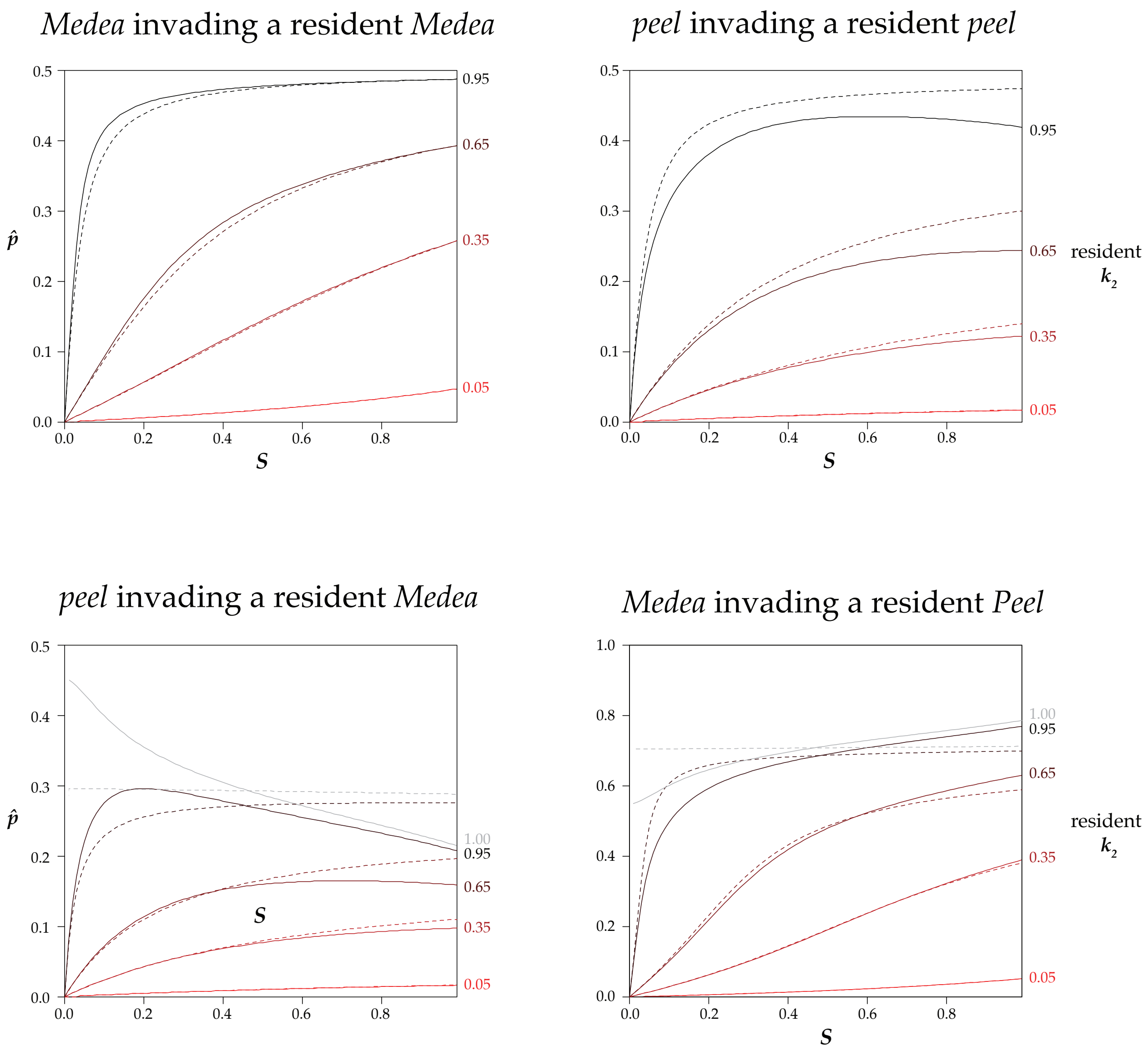

Supplement: 2 [file NIHPP2024.07.23.604817v1-supplement-2.pdf]
